# Preclinical experience with a novel single-port platform for transoral surgery

**DOI:** 10.1007/s00464-021-08420-2

**Published:** 2021-03-12

**Authors:** Emily K. Funk, Philip Weissbrod, Santiago Horgan, Ryan K. Orosco, Joseph A. Califano

**Affiliations:** 1 Division of Head and Neck Surgery, Department of Surgery, University of California San Diego, San Diego, CA USA; 2 Division of Minimally Invasive Surgery, Department of Surgery, University of California San Diego, San Diego, CA USA; 3 Division of Otolaryngology – Head and Neck Surgery, Department of Surgery, Moores Cancer Center, University of California San Diego, 3855 Health Sciences Drive, La Jolla, CA 92093 USA

**Keywords:** Transoral surgery, Minimally invasive surgery, Head and neck surgery, Laryngeal microsurgery

## Abstract

**Background:**

We investigated a novel minimally invasive surgical platform for use in the oropharynx, hypopharynx, and larynx for single-port transoral surgery used in concert with standard transoral laryngeal and pharyngeal instrumentation.

**Methods:**

The preclinical investigational device by Fortimedix Surgical B.V. (Netherlands) features two channels for manually controlled flexible articulating surgical instruments. A third central channel accepts both rigid and flexible endoscopes. The system is coupled to a standard laryngoscope for transoral access. In three cadaver models, we evaluated the surgical capabilities using wristed grasping instruments, microlaryngeal scissors, monopolar cautery, and a laser fiber sheath. Procedures were performed within the oropharynx, supraglottis, glottis, subglottis, and hypopharynx.

**Results:**

Within the oropharynx, we found adequate strength, range of motion, and dexterity to perform lateral oropharyngectomy and tongue base resection. Within the larynx, visualization was achieved with a variety of instruments including a flexible, 0° and 30° rigid endoscope. The glottis, supraglottis, pyriform sinuses, post-cricoid space, and esophageal inlet were readily accessible. Visualization and manipulation of grasping, laser, and monopolar cautery instruments were also possible within the subglottis. Instrument reach and accuracy facilitated completion of a delicate micro-flap on the true vocal fold. Other procedures included vocal fold resection, cricopharyngeal myotomy, and resection of subglottic mucosa.

**Conclusions:**

From this initial proof of concept experience with this novel platform, we found a wide range of procedures within the oropharynx, larynx, and hypopharynx to be feasible. Further work is needed to evaluate its applicability to the clinical setting. The ability of this platform to be used with conventional instrumentation may provide an opportunity for complex transoral surgery to be performed in a facile manner at greatly reduced cost.

**Supplementary Information:**

The online version contains supplementary material available at 10.1007/s00464-021-08420-2.

Transoral approaches to benign, malignant, and structural disorders of the upper aerodigestive tract are limited. Traditional surgical technologies utilize line-of-site visualization, via microscope or telescope, and rigid instrumentation. These are less than ideal solutions for operating in a non-linear, narrow, funnel-shaped anatomic space.

Robotic technologies have seen expanded indication in this anatomic region with the advent of smaller instrumentation. The most common current head and neck application of robotic technology is for disease of the oropharynx which includes the soft palate, tonsils, and base of tongue. In recent years, with the increase in HPV-mediated oropharyngeal disease [1, 2], there has been rise in utilization of transoral robotic surgery (TORS), as surgical resection alone can be used to treat select oropharyngeal cancers effectively [3]. Robotic surgery can provide excellent access to regions that are not easily visualized with line of site but still lie within proximity to the opening of the oral cavity.

The larynx, located more distal, includes the epiglottis, arytenoids, false vocal folds, and true vocal folds. Robot use is limited in this region due to narrow anatomic confines. Intervention typically relies more heavily on transoral rigid visualization and instrumentation. The use of rigid instrumentation presents some technical challenges given the long distance from instrument fulcrum to tissue which can lead to exaggerated physiologic tremor. Range of motion and dexterity are also reduced given the narrow working space as the instruments pass through the laryngoscope. In addition to these challenges, the fine motor control mandated by rigid endoscopes can be problematic. The hypopharynx and upper esophageal sphincter, otherwise known as the pharyngoesophageal segment (PES), have similar limitations in regard to surgical access [4–6]. TORS is used only for treatment of select malignancies of the hypopharynx and larynx [7–9] when access is feasible.

Despite the enhanced surgical capabilities, user access to robotic surgery can be limited due by the high startup costs of the system. In general, the initial cost of a robotic platform is approximately 1.5 million dollars, with significant additional costs for annual maintenance and support, instruments, staff training, and increased operating room times [10, 11]. While minimally invasive treatment using TORS has been shown to decrease length of hospital stay and hospital-related costs, initial evaluations show that TORS remains more expensive than standard approaches [12, 13].

Here, we investigate a novel minimally invasive surgical platform for its utility in the transoral approach to the oropharynx, hypopharynx, and larynx. The Fortimedix Surgical B.V. symphonX™ single-port surgical platform is FDA approved for minimally invasive abdominal laparoscopic surgery and has been described for use in laparoscopic cholecystectomy [14, 15]. This platform has been adapted from a single-port laparoscopic device and modified to allow introduction via a surgical laryngoscope for use in the transoral approach to the upper aerodigestive tract. It is designed for applications similar to that of the Flex® Robotic System (Medrobotics, Raynham, MA) and da Vinci Single Port (Intuitive, Sunnyvale, CA) platforms [16–20]. However, its simplicity of design may facilitate lower cost allowing for greater value and potentially improved access in low resource settings within a wide variety of surgical applications.

## Materials and methods

**Fig. 1:**
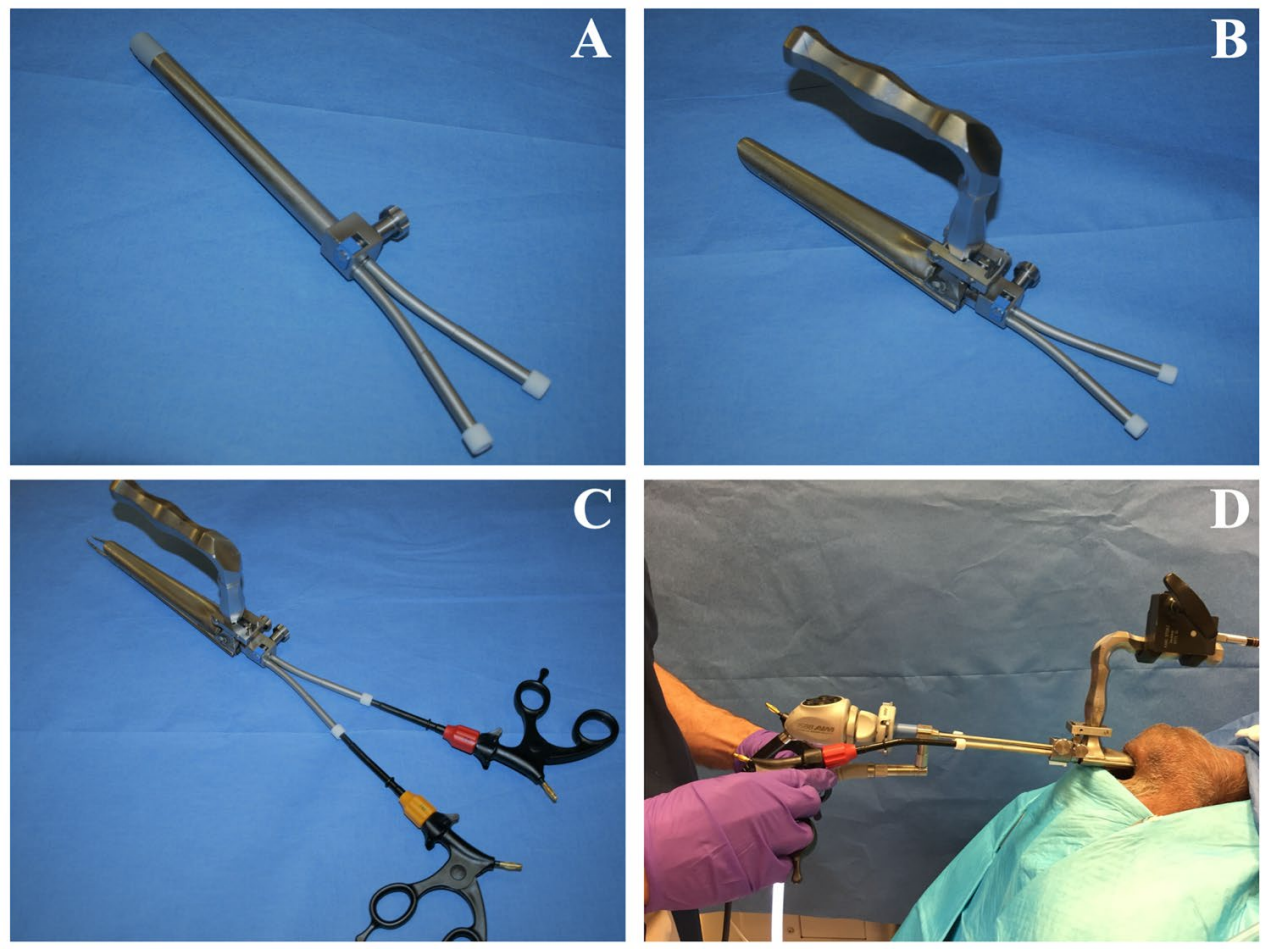
**A** Single-port device **B** Jako laryngoscope with device inserted **C** Wristed instruments inserted into instrument channels **D** Bedside setup with rigid endoscope in place

This investigation was limited to cadaveric specimens and as such did not require IRB review. The preclinical investigational device by Fortimedix Surgical B.V. was originally developed for abdominal laparoscopic surgery. It has been adapted for use as a minimally invasive platform for transoral surgery by specific modifications in design. This platform features a re-usable introducer device that is inserted transorally in conjunction with standard laryngoscopes for surgical access to the oropharynx, hypopharynx, and larynx (Fig. 1), single-use flexible, wristed articulating surgical instruments, and re-usable instrument handles.

The introducer device (Fig. 1A; from here forward referred to as “the introducer”) is 14 mm in diameter and contains three working channels that course through it. The introducer is 160 mm in length. The central channel measures 5.5 mm in diameter and accepts either rigid or flexible laryngeal endoscopes. Two 4.5 mm instrument channels run along either side of the endoscope channel.

This platform features manually controlled, flexible, wristed articulating instruments (Fig. 1C) including grasping forceps, micro-forceps, micro-scissors, hook monopolar cautery, and a fiberoptic laser manipulator. Instruments are 3.4 mm in diameter with a working length of 230 mm (total length 390 mm). The instruments are designed for single use, while the introducer and instrument handles are re-usable. The fiberoptic laser manipulator is compatible with KTP or CO2 laser systems. Instrument architectural design is unique in that there are no traditional pulling wires for articulation. Rather, the articulating instruments are built with multiple layers of laser cut tubes that are assembled and welded together to support each other and establish overall instrument functionality. This tubular design allows for multi-directional end-effector response and improved haptic response and durability. Additionally, 360-degree rotation is achieved by turning the rotation knob on the re-usable handle. The materials and laser cut design allow for production of flexible, wristed instruments with low production costs.

The introducer is inserted through the oral cavity after placement of a standard mouth retractor or laryngoscope. After insertion, it can be suspended independently from the laryngoscope through use of a suspension arm that attaches to the surgical table. Alternatively, the device can be coupled directly to the laryngoscope to secure it in place (Fig. 1B). The direct coupler is available for use with a Lindholm laryngoscope (Karl Storz, El Segundo, CA) for procedures within the oropharynx or to a Jako or Dedo laryngoscope (Pilling, Morrisville, NC) for hypopharyngeal and laryngeal procedures. A modified introducer with a curved structure is also available, which requires the use of a flexible laryngoscope.

Surgical setup is largely analogous to traditional laryngoscopic procedures. An appropriate laryngoscope is selected, placed, and suspended in standard fashion. The device slides into the laryngoscope frame and is secured in place; an endoscope is introduced through the central channel. The laryngoscope can be repositioned or adjusted as needed after the device is attached to obtain optimal visualization. Wristed instruments are then placed into left- and right-sided working channels and can be exchanged throughout the procedure (Fig. 1D). In our experience, once the laryngoscope was suspended, placement of the device and positioning of the endoscope and instruments took less than five minutes. Once setup is complete, the surgeon can choose to stand or sit at the head of the bed; endoscope monitors can be positioned over the patient’s chest.

The study was performed in three separate operative sessions; an adult cadaver head was used in the first session and an adult bell torso cadaver head was used in sessions two and three. The first session we performed a lateral oropharyngectomy and base of tongue resection with the use of the standard off-the-shelf Fortimedix Surgical B.V. symphonX™ surgical platform for abdominal laparoscopic surgery, featuring 5 mm articulating instruments, in concert with a Feyh-Kastenbauer retractor (Gyrus Medical, Tuttlingen, Germamy). The StrongArm retractor (Mediflex, Islandia, NY) was used with the symphonX™ introducer device suspended by framework secured to the surgical table. A 0-degree 5 mm rigid endoscope was used in conjunction with the 5 mm symphonX™ grasper and hook monopolar cautery articulating instruments. We performed a preliminary evaluation of the device and setup in the operating suite. During the second session, we performed a preliminary evaluation of the investigational device for feasibility in regard to accessing the esophageal inlet and tracheolaryngeal complex. No surgical procedures were performed.

During the first and second sessions with the investigational device, we found that using a separate frame to suspend the introducer separately from the laryngoscope was somewhat cumbersome. Independent positioning and placement of the introducer did not seem to provide significant benefit. This was discussed with the developers and device modifications were made in preparation for the third session, which proved to be crucial to the success of further work.

During the third session, we performed the following procedures with the investigational device: subepithelial cordectomy (type I ELS), transmuscular cordectomy (type III ELS), partial (supraglottic) laryngectomy, cricopharyngeal myotomy, and subglottic resection of focal lesion. A Jako laryngoscope and 0-degree 5 mm rigid endoscope were used for exposure of the hypopharynx and larynx. The device modifications included addition of an adaptor that attached the introducer to the laryngoscope itself, eliminating the need for a separate suspension frame. We found that this modification effectively coupled the introducer and the laryngoscope, improving ease of setup and decreasing equipment burden at the head of the bed.

## Results

### Oropharynx

**Fig. 2:**
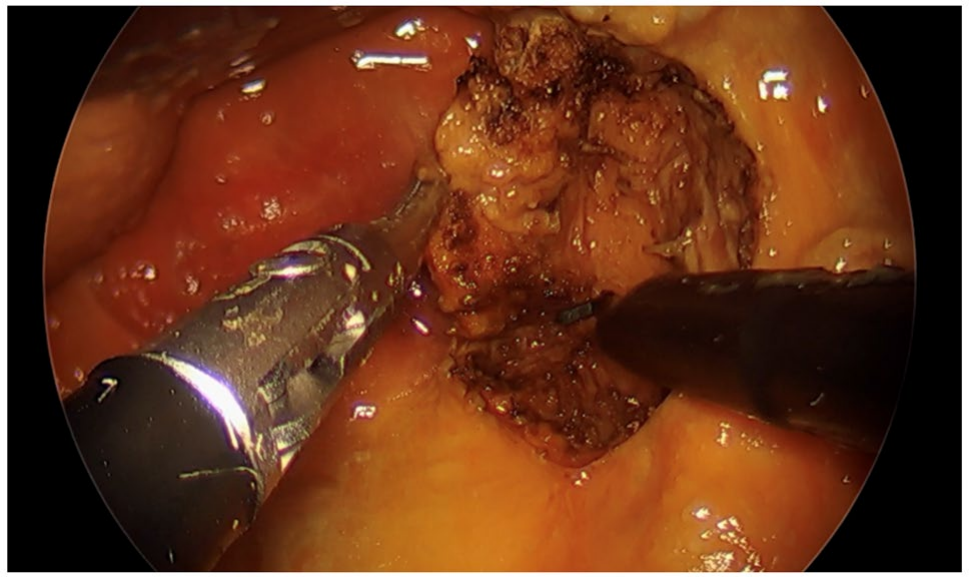
Lateral oropharyngectomy performed with monopolar cautery hook

Oropharyngeal procedures were performed in the first session using a partially dentulous cadaver. We performed the setup as described above using a 0-degree 5 mm rigid endoscope. We performed both lateral oropharyngectomy and base of tongue resection as described in robotic literature [20, 21] using the flexible grasper and hook monopolar cautery instruments (Fig. 2). A bedside assistant was present to provide smoke evacuation.

We found visualization and access to the soft palate, lateral oropharynx, and base of tongue were comparable to robotic platforms. The instruments provided the required strength for retraction of the tissues of the oropharynx. The dexterity and range of motion were comparable or exceeded those of a robotic system. No tremor was noted during these procedures. The use of manual instruments was dissimilar to the manipulation of the robotic instrument controls of the daVinci platform (Intuitive Surgical, Sunnyvale, CA). While this style of manual instrumentation is well known to laparoscopic surgeons in other fields, it was noted by our otolaryngologic surgeons as intuitive and comfortable (Table 1).

**Table 1:** Characteristics of transoral surgery modalities

	Direct (endoscopic/microscopic)	da Vinci Xi and SP	Medrobotics Flex®	Fortimedix symphonX™
Endoscope	Rigid/ Microscope	Rigid/Flexible (SP)	Flexible	Rigid/Flexible
3D – capable	Binocular microscope	Yes	With surgeon glasses	No
Setup	Simple	Complex	Moderate	Simple
Cost	Low	High	High	Low
Instruments	Rigid	Wristed	Wristed	Wristed
Dissecting instrument	Yes	No	No	Yes
Micro-instruments	Yes	No	Yes	Yes
Laser compatible	Yes	No	Yes	Yes
Tremor reduction	No	Yes	Yes	Yes
Scaling	No	Yes	No	No
Haptic feedback	Yes	No	Partial	Partial
Crowding in surgical field	Yes	Yes (reduced with SP)	Decreased	Decreased
Crowding at head-of-bed	No	Yes	Yes	No
Oropharynx access	Limited	Yes	Yes	Yes
Hypopharynx access	Yes	Limited/ Difficult	Yes	Yes
Larynx access	Yes	Limited/ Difficult	Yes	Yes

In contrast to the robotic platforms typically used for these procedures, we found several advantageous features of this device within the oropharynx. The setup of this device was simple and quick, particularly given the use of equipment commonly used in an otolaryngologic operating room. The compact and streamlined design of the device allowed for minimal crowding of the head of the bed, with easy access for a bedside assistant. Given the use of manual instrumentation, haptic feedback was appreciated. There were no major obstacles noted specific to work within the oropharynx.

### Larynx

During the third session, glottic procedures were performed using a partially dentulous cadaver. An endotracheal tube was placed under direct visualization to reflect the standard surgical environment. The introducer and laryngoscope were set up as described. A Jako laryngoscope and 0-degree 5 mm rigid endoscope were used. Visualization of the glottis was obtained with adequate view of bilateral true and false cords, anterior commissure, and subglottic space.

**Fig. 3:**
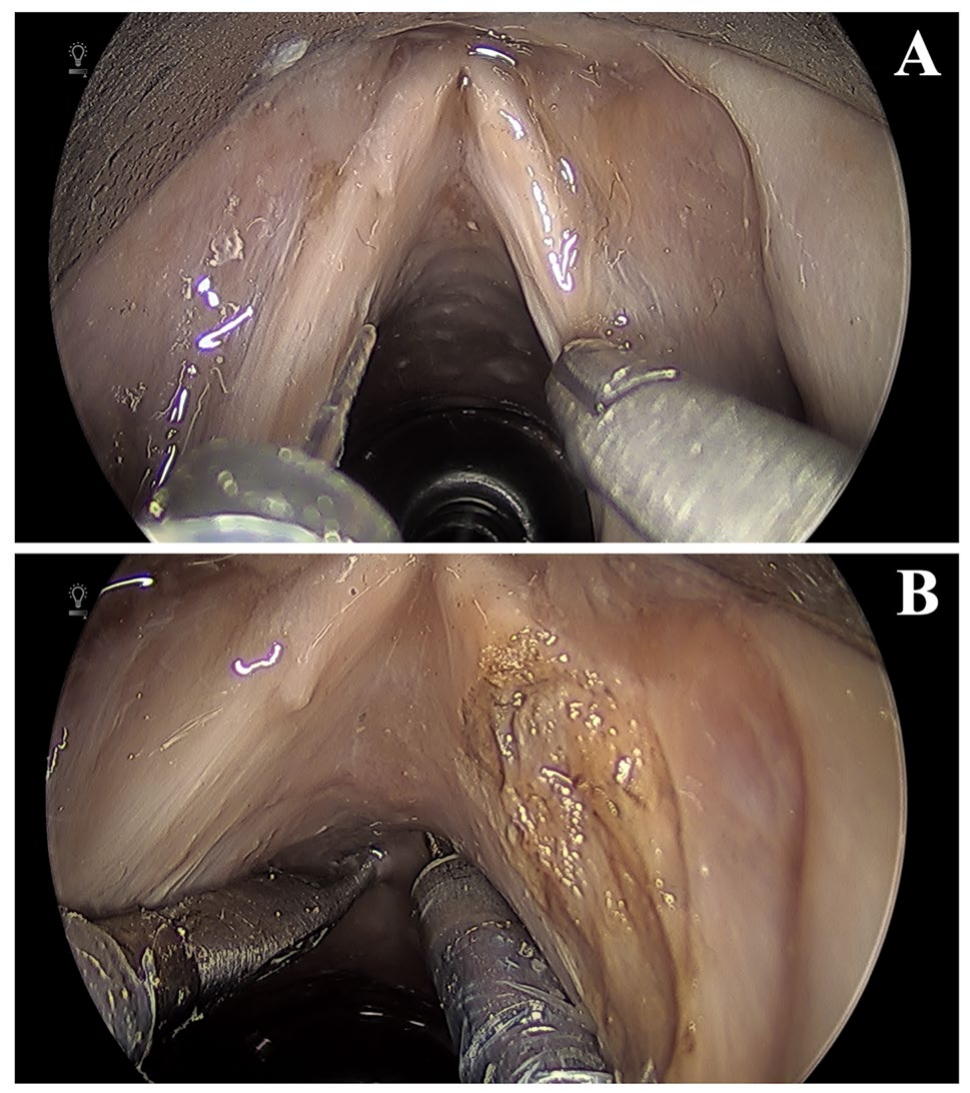
**A** Fiberoptic laser manipulator (left) and micro-forceps with visualization of the glottis **B** Demonstration of subglottic reach

An Endostat 0.6 mm (Boston Scientific, Marlborough, MA) glass fiber, typically used with a KTP laser, was inserted into the fiberoptic laser manipulator instrument. While no laser was available for use, we evaluated range of motion, dexterity, and access to difference laryngeal structures (Fig. 3A). Despite the narrow access afforded by a laryngoscope, the wristed endoscopic instruments allowed for enhanced range of motion in all directions beyond our current rigid laryngeal instruments. Access distally into the infraglottic and subglottic regions was also possible without comprise precision or control of movement (Fig. 3B).

**Fig. 4:**
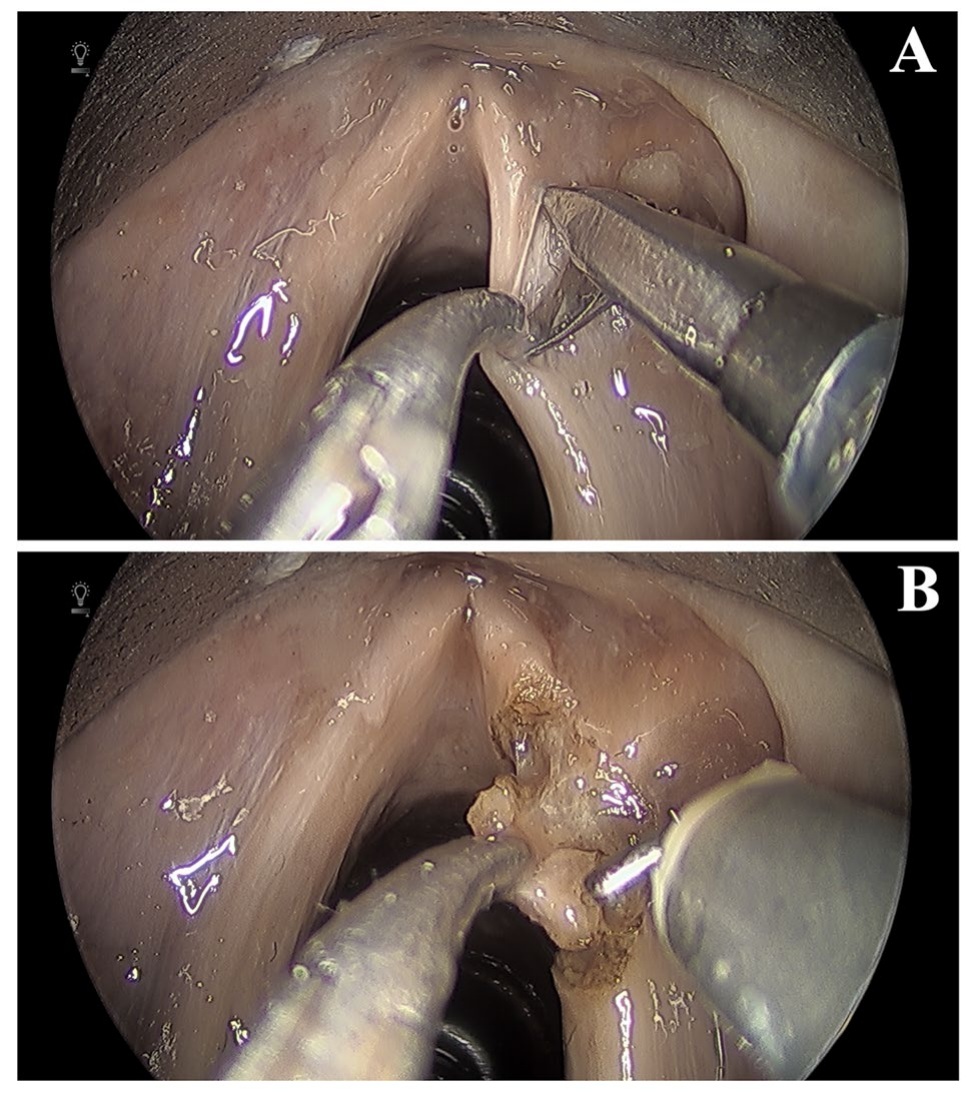
**A** Micro-scissors (right) shown elevating micro-flap in subepithelial cordectomy **B** Monopolar cautery hook (right) shown performing transmuscular cordectomy

The fiberoptic laser manipulator was replaced with the micro-scissor instrument. A subepithelial cordectomy or type I European Laryngological Society (ELS) was performed. A micro-flap was elevated from the true vocal fold with use of the micro-scissors for dissection to separate tissue planes (Fig. 4A). Subsequently, the micro-forceps and hook monopolar cautery instruments were used to perform a transmuscular (type III ELS), involving excision of the epithelium, Reinke’s space, vocal ligament and thyroarytenoid muscle (Fig. 4B). We found visualization and manipulation of the instruments allowed for excellent tissue control facilitating accurate resection. The same instruments were then used to perform subglottic resection of focal lesion. There was no compromise in control of the instruments with this distal work.

During these glottic procedures, we found that the surgical setup and visualization were analogous to traditional microlaryngeal procedures. However, the wristed instruments provided dexterity and improved range of motion. The instrument size was appropriate for delicate work, and no compromise to strength was noted. The features specifically advantageous to glottic work included the significant opening strength of the scissors for tissue dissection, as well as haptic feedback and lack of tremor. The platform also performed well in distal work in the subglottis.

While working in the glottic region was largely successful, currently, the breath of available instruments is limited. Traditional rigid laryngeal trays include many unique instruments that are tailored to the specific procedures in this region. The development of unique instruments for novel platforms is a lengthy process, and initial application of this platform will be constrained by available instrument options. Also, the Jako laryngoscope tends to be on the larger spectrum of laryngeal scopes and may not be able to be placed in all patients.

### Hypopharynx

**Fig. 5:**
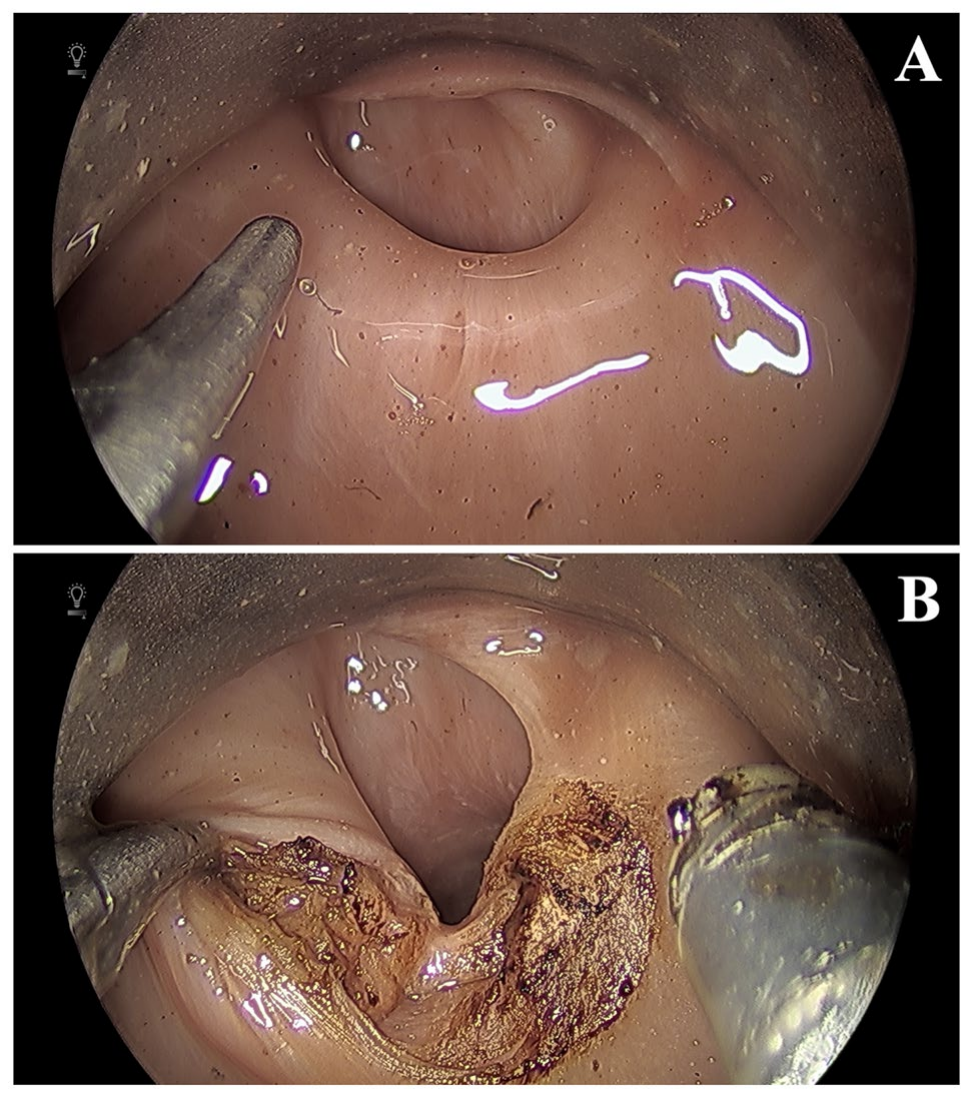
**A** Cricopharyngeus muscle and esophageal inlet **B** Transection of cricopharyngeus muscle with monopolar cautery hook

The platform was also investigated for work in the hypopharynx. The laryngoscope was repositioned for access to the pyriform sinuses, post-cricoid space, and esophageal inlet. A cricopharyngeal myotomy was performed using the micro-forceps and hook monopolar cautery (Fig. 5). Again, despite a relatively narrow surgical field, the instruments allowed for excellent control of the tissues and short procedure time. While we successfully performed the cricopharyngeal myotomy with cautery, it would be feasible to perform this procedure with the fiberoptic laser manipulator as well.

The primary advantage of using this platform in the hypopharynx is the ability to obtain visualization that cannot be achieved with larger robotic platforms and features flexible instrumentation to accomplish surgical tasks that are not feasible with the rigid instruments. This design combines the advantageous features of traditional visualization with a laryngoscope with the dexterity and freedom of movement of wristed instrumentation.

We expect the challenges of using this platform in the hypopharynx will be those that are inherent to transoral work in this space. In particular, larger lesions and some anatomic phenotypes present access limitations for any form of rigid endoscopic equipment.

## Discussion

Traditional transoral approaches to the upper aerodigestive tract rely on direct line-of-site visualization and linear rigid instrumentation. Within the larynx, the use of a laryngoscope in conjunction with a microscope or endoscope allows for visualization of desired subsites of the endolarynx and hypopharynx. In fact, the placement and positioning of a laryngoscope for visualization are an essential skill learned in training which continues to develop with experience. While a large array of specialized rigid laryngeal instruments exists, the skillful application of these instruments is technically challenging given the narrow working confines of a laryngoscope, which can lead to challenges with precision, maneuverability, and visualization. In addition, the long lever arm of the instruments causes magnification of tremor, which is further exaggerated under microscopic view. Nonetheless, these approaches remain the mainstay for work within the endolarynx.

The introduction of transoral robotic surgery has allowed for improved visualization of and access to select subsites of the upper aerodigestive tract, particularly the oropharynx. In TORS, a midline rigid endoscopic camera is introduced transorally along with articulating instrument arms. The most notable advantage of robotic technology over traditional transoral approaches is the substitution of rigid instruments for flexible ones. While this approach has been most successful for procedures in the oropharynx, robotic platforms are rarely applied to surgical procedures in the hypopharynx and larynx. Robotic platforms are challenged in their application to the upper aerodigestive tract due to issues with the angle of approach, device footprint, and instrument crowding. The need for miniaturization of robotic instruments is an additional barrier to applications in the endolarynx.

Advances have been made in recent years to specialize robotic platforms for use in the upper aerodigestive tract. Anatomic limitations of this region remain the origin of challenges with access; however, novel technologies have attempted to solve this issue by using a non-linear approach. Newer platforms such as the Medrobotics Flex® Robotic System and da Vinci Single Port platforms employ flexible endoscopic devices and instrumentation within a condensed single sheath. This eliminates the bulk and difficulty associated with placement of multiple arms through the oral opening and allows access and maneuverability in a non-linear, snake-like fashion. These iterations are tailored to the transoral approach and are becoming increasingly utilized for transoral robotic procedures.

While robotic technologies are constantly being improved, these platforms are associated with significant costs. While the initial price point of investing in the device itself is typically over a million dollars, additional costs include annual maintenance and support fees, instrument costs, infrastructure upgrades, staff training, and increased operating room times. While robotic platforms tailored to the head and neck are particularly appealing to an otolaryngologist, the utilization of the platform by a single surgical subspecialty makes the cost difficult to justify, except in hospitals with large otolaryngology groups. Ultimately, the cost of these platforms is prohibitive for many institutions.

The Fortimedix surgical platform is evaluated in this preclinical study as a lower cost alternative for transoral minimally invasive surgery. In our experience with this platform, we found the design of the device contributed to ease in setup, as it is used with equipment well known to otolaryngologists. The platform is both laryngoscope and endoscope agnostic, allowing the surgeon to select their preferred laryngoscope and endoscope for each surgery. This also provides flexibility in the selection of the laryngoscope in order to obtain the preferred visualization, which can be further augmented with use of a flexible endoscope if needed.

The small footprint of this platform provides a working environment dramatically less crowded than that of TORS, essentially analogous to that of a direct laryngoscopy. The size of the introducer was favorable, given the diameter of 14 mm. The transoral component of the daVinci Single port measures 25 mm in diameter, while that of the Flex® Robotic System is 18 mm in height (28 mm in width with instrument channels). We expect this will provide ease of insertion in patients with limited oral opening, as this is considerably smaller than platforms we use currently. Elimination of overall size of the device also seems to contribute to increased reach, as demonstrated by our ability to perform an excision within the subglottis.

Instrumentation featured improved range of motion relative to rigid instruments and similar to robotic instruments. Both the 5-mm and 3.4-mm diameter instruments were used in these dissections. While tissue handling was precise and delicate, an additional notable advantage of this platform was the opening strength of the instruments, which allowed for dissection and spreading of tissue planes. The use of manual flexible instruments offered both tremor reduction and haptic feedback, reminiscent to that of the Flex® Robotic System. The ability to select and utilize a laser system for use with the laser fiber manipulator is favorable, given the frequency in which laser systems are utilized in the treatment of structural disorders of the upper aerodigestive tract.

Throughout the study, we identified some areas that may require improvement with continued development. One limitation of this platform relative to robotic platforms is the lack of 3D visualization, which provides excellent depth perception within the surgical field. While otolaryngologists are accustomed to working with endoscopic visualization, the depth perception provided by a robotic platform or microscope is highly advantageous and would confer additional benefit if implemented in this platform. Potential adaptations would include use of three-dimensional rigid endoscopic systems within the platform.

Access issues are similar to those encountered using standard surgical laryngoscopes. The Jako and Lindholm tend to be on the larger end of the spectrum in regard to working space. In cases where laryngoscopic access is challenging, the device may not be useable in its current form. Further scaling would allow for more broad application, in particular in challenging laryngeal and hypopharyngeal exposures.

We found that the precision of the instruments was similar to that of the DaVinci for oropharyngeal applications but significantly enhanced in microlaryngeal instrumentation, due to lack of tremor, ability to precisely control scissors opening, and adequate opening strength to perform delicate dissection. In our experience, the learning curve required to adjust to the characteristics of manually controlled instruments is minimal. Additionally, their functionality, practicality, and lower cost provide an exciting alternative for expanding our use of transoral minimally invasive procedures.

Further development of additional instruments will increase applications for a variety of surgical procedures. In particular, the platform does not have a needle tip device for injections, which are commonly used in endolaryngeal procedures. The hook monopolar was sufficient in our work, but availability of a needle and spatula monopolar instrument is needed given the specific preferences of different surgeons. While some additional instrumentation will likely be developed over time, including bipolar instruments, curved scissors, needle drivers, tissue elevators, and suction irrigation devices, others may be inherently limited due to the use of manual instruments. A 5-mm clip applier instrument is available but was not evaluated in this study. The daVinci system offers a variety of standard instruments as well as more sophisticated instruments including tissue sealing forceps, stapling devices, and clip appliers. These types of instruments are not available on the Flex® Robotic System or Fortimedix platform at present. However, the engineering design of such complex instrumentation is inherently challenging for manual instrumentation.

A final consideration in the evaluation of this platform is that of cost. As discussed, costs of robotic procedures can be prohibitive for many hospitals [10]. The Fortimedix surgical platform has the potential to be dramatically less costly, particularly given the utilization of instrumentation already available in any otolaryngologic operating suite. In this manner, the platform has the potential to be more accessible, expanding access for both patients and surgeons.

## Conclusion

From this initial experience, using the Fortimedix Surgical minimally invasive platform, we found a wide range of procedures within the oropharynx, larynx, and hypopharynx to be feasible. Initial use of this platform in the cadaveric setting revealed that it was feasible for use in procedures currently performed using transoral robotic surgery, as well as some distal procedures currently performed using transoral laser microsurgery or transoral endoscopic surgery. The hybridization of flexible instruments similar to that of robotic platforms with new framework that can be applied to traditional laryngoscopes and endoscopes presents a promising new alternative for transoral surgery. Further work is needed to evaluate its applicability to the clinical setting.

## Supplementary Information

Below is the link to the electronic supplementary material.

Supplementary file1 (MP4 49367 KB)

Supplementary file2 (MP4 57851 KB)

Supplementary file3 (MP4 70411 KB)

Supplementary file4 (MP4 45082 KB)
